# Hyperpolarised NMR to aid molecular profiling of electronic cigarette aerosols†

**DOI:** 10.1039/d1ra07376a

**Published:** 2022-01-10

**Authors:** Ben. J. Tickner, Sanna Komulainen, Sanna Palosaari, Janne Heikkinen, Petri Lehenkari, Vladimir V. Zhivonitko, Ville-Veikko Telkki

**Affiliations:** NMR Research Unit, Faculty of Science, University of Oulu 90014 Finland vladimir.zhivonitko@oulu.fi ville-veikko.telkki@oulu.fi; Cancer and Translational Medicine Research Unit, Faculty of Medicine, University of Oulu 90014 Finland; Medical Research Center Oulu, Faculty of Medicine, University of Oulu and Oulu University Hospital 90014 Finland; Division of Orthopedic Surgery, Oulu University Hospital 90220 Finland

## Abstract

Signal amplification by reversible exchange (SABRE) hyperpolarisation is used to enhance the NMR signals of nicotine and acrolein in methanol-d_4_ solutions of electronic cigarette aerosols. Consequently, detection of 74 μM nicotine is possible in just a single scan ^1^H NMR spectrum. The first example of an aldehyde hyperpolarised using SABRE is demonstrated and we work towards novel real-world applications of SABRE-hyperpolarised NMR for chemical analysis.

## Abstract 2

SABRE hyperpolarisation enhances NMR signals of low concentration nicotine and acrolein and allows quantitative detection in electronic cigarette aerosol solutions.

## Graphical abstract

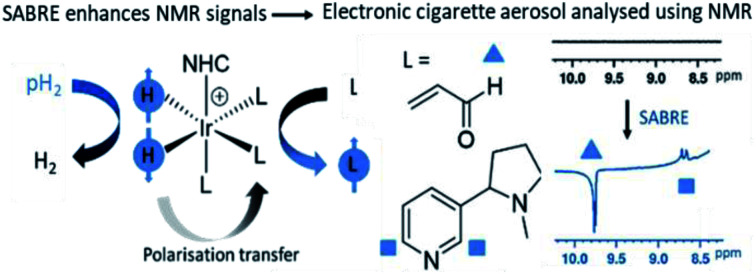

## Introduction

Electronic cigarette aerosols are becoming increasingly popular with an estimated 41 million users as of 2018.^1,2^ Unlike traditional cigarettes that involve tobacco combustion, electronic cigarettes heat and vapourise cigarette fluid. The composition of these inhaled aerosols, which are often studied using techniques such as Gas Chromatography Mass spectrometry (GC-MS) or Nuclear Magnetic Resonance (NMR) is not fully understood.^1,2^ A greater understanding of the chemical composition of these aerosols, and their solutions, is essential given the role they may play in user's health. NMR is used routinely for molecular identification and is often the method-of-choice for many scientists as it does not use ionising radiation or destroy the sample.^3^ However, NMR experiments are typically repeated many times to generate discernible signals due to the ppm thermal polarisation of NMR active nuclei.^4^ This low sensitivity can often make NMR experiments time consuming and necessitates the use of concentrated samples (>*ca.* 1 mM) for analysis by NMR.

**Fig. 1:**
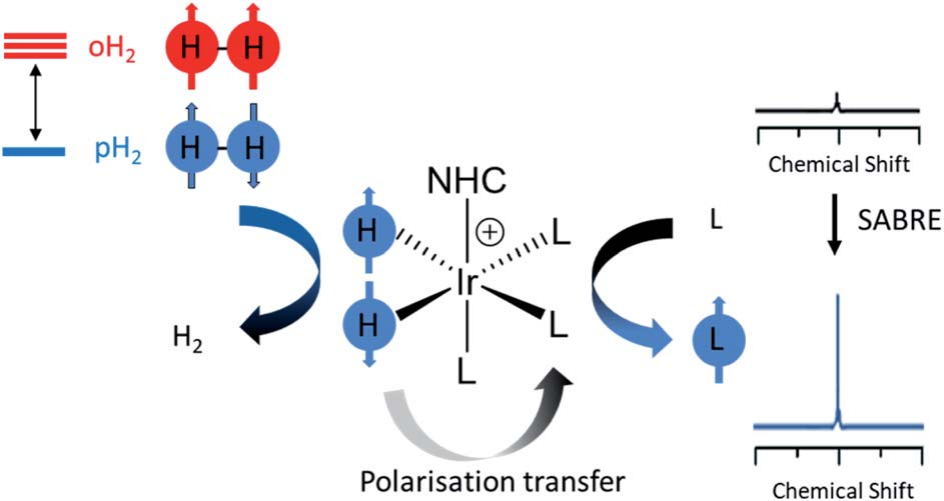
Depiction of SABRE. Hydrogen exists as 75% *ortho*hydrogen (*o*H_2_) and 25% *para*hydrogen (*p*H_2_) at room temperature; it can be enriched in the *para* isomer by cooling to low temperature. SABRE uses reversible oxidative addition to unlock latent magnetism within *p*H_2_. A target ligand can become hyperpolarised following reversible exchange with the SABRE catalyst. Consequently, its NMR signals can be enhanced by many orders of magnitude.

Nuclear spin hyperpolarisation can generate NMR signals enhanced by many orders of magnitude compared to those recorded using thermal polarisation.^4^ Signal Amplification By Reversible Exchange (SABRE) is one such hyperpolarisation technique that uses *para*hydrogen (*p*H_2_) as its source of enhanced magnetisation, which is a spin isomer of dihydrogen that is cheap and easy to produce. The latent magnetism of *p*H_2_ can be unlocked and transferred to a target molecule *via* reversible interactions with a metal catalyst.^5^ Polarisation transfer occurs *via* a temporary *J*-coupled network that exists within the short-lived SABRE catalyst (Fig. 1).^6,7^ SABRE is a low-cost route to produce molecules in an enhanced nuclear spin state. However, hyperpolarisation soon decays back to thermal equilibrium according to *T*_1_ relaxation and must therefore be measured rapidly. The reversibility of SABRE provides an additional benefit as these enhanced NMR signals are easily regenerated upon fresh shaking or bubbling with *p*H_2_.^5,8^ SABRE has been reported to hyperpolarise a wide range of molecular targets such as N-donor ligands^9^*via* catalysts of the form [Ir(H)_2_(NHC)(substrate)_3_]Cl where NHC is an N-heterocyclic carbene. SABRE has produced ^1^H, ^15^N, and ^13^C polarisation of 65%,^10^ 79%,^11^ and 4%^12^ respectively, and NMR signals of other heteronuclei have also been enhanced.^13–18^ SABRE-enhanced NMR signals have been involved in a broad range of applications including reaction monitoring,^19,20^ mechanistic elucidation,^21^ magneto-optics^22^ and many others.^23^ In particular, use of SABRE-hyperpolarised NMR for mixture analysis is promising as it provides a method for boosting the NMR signals of many low concentration analytes.^24–30^ In this work, the NMR sensitivity improvements that SABRE hyperpolarisation can provide are employed to aid detection of low concentration analytes in electronic cigarette aerosols dissolved in methanol-d_4_ using NMR.

## Results and discussion

Two samples of electronic cigarette aerosol were prepared and studied: Ecig contained electronic cigarette fluid with 18 mg mL^−1^ nicotine which was vapourised and dissolved in methanol-d_4_ (see ESI, Section S1.2†). A background reference sample, Ecig^diluent^, contained dissolved aerosol from electronic cigarette carrier fluid without nicotine. Thermally polarised ^1^H and ^13^C NMR measurements of these aerosol solutions (0.1 mL) in methanol-d_4_ (0.5 mL) at 298 K and 9.4 T were able to confirm the presence of a wide range of molecules including acetaldehyde, propylene glycol, glycerol, hydroxyacetone, 1-propenol, and formaldehyde (see ESI, Section S2†). These molecules have all been identified previously in electronic cigarette aerosols using LCMS, GCMS, or NMR spectroscopy.^1,2^ In these concentrated solutions, these molecules are all present at concentrations *ca.* >5 mM and these thermally polarised measurements require *ca.* 20 min to acquire. In this work we investigate the use of SABRE-hyperpolarisation to enhance NMR signals for analytes of interest, such as nicotine, and use this sensitivity boost to aid chemical analysis of electronic cigarette aerosol solutions when analyte concentrations are much lower than mM levels.

The enhancement of ^1^H NMR signals for the N-heterocycle nicotine, which is present in many tobacco products,^31^ has been reported under many different conditions elsewhere.^5,29,32–35^ We reacted SABRE precatalyst [IrCl(COD)(IMes)] (5 mM) (where COD is *cis*,*cis*-1,5-cyclooctadiene and IMes is 1,3-bis(2,4,6-trimethyl-phenyl)imidazol-2-ylidene) and (−)-nicotine (5 equiv.) with H_2_ (3 bar) overnight at room temperature in methanol-d_4_ (0.6 mL) and confirmed the formation of the active catalyst [Ir(H)_2_(IMes)(nicotine)_3_]Cl by 2D NMR characterisation (see ESI, Section S3.2†). These solutions were shaken vigorously with *p*H_2_ (3 bar) for 10 seconds to yield enhanced ^1^H NMR signals at *δ* = 8.52, 8.46, 7.45 and 7.88 ppm corresponding to the two inequivalent *ortho*, *meta* and *para* resonances of the free nicotine pyridine ring respectively. These are 66 ± 9, 65 ± 12, 107 ± 19 and 87 ± 17 times larger than those recorded under Boltzmann conditions respectively (see ESI, Fig. S12†). In our experiments, this shaking process was performed inside an electromagnetic coil that generates a magnetic field of *ca.* 6.5 mT (see ESI, Section S1.4†).^36^ This field was selected as it has been reported optimal for most efficient SABRE polarisation transfer to ^1^H sites within nicotine and similar N-heterocyclic targets.^5,37^ After shaking, samples were rapidly inserted into a 9.4 T NMR spectrometer for collection of a single scan ^1^H NMR spectrum.

We turn our attention to the detection of small concentrations (<1 mM) of nicotine, rather than the *ca.* 25 mM substrate concentrations typically used in SABRE measurements, or the *ca.* 6 mM nicotine concentrations in our electronic cigarette solutions analysed using thermally polarised NMR. For these experiments, the inclusion of a coligand is essential to allow formation of a stable SABRE catalyst of the form [Ir(H)_2_(IMes)(coligand)_3_]Cl. Trace analytes can then displace a coligand to form [Ir(H)_2_(IMes)(analyte)(coligand)_2_]Cl which catalyses polarisation transfer from *p*H_2_ to an analyte giving rise to enhanced analyte NMR signals. This approach facilitates detection of substrate concentrations lower than that of the catalyst as the substrate is no longer required in at least a 3-fold excess relative to the metal centre.^24,26^ It has also been reported that the hyperpolarised NMR signal intensity of dilute substrates in the presence of a coligand can be proportional to their concentration due to the formation of small amounts of [Ir(H)_2_(NHC)(coligand)_2_(analyte)]Cl relative to [Ir(H)_2_(IMes)(colignad)_3_]Cl.^24,26^ Standard additions of known analyte concentrations are made to a sample of interest and the linear increase in hyperpolarised analyte signal is used to determine the original analyte concentration prior to spiking.^24,26^

**Fig. 2:**
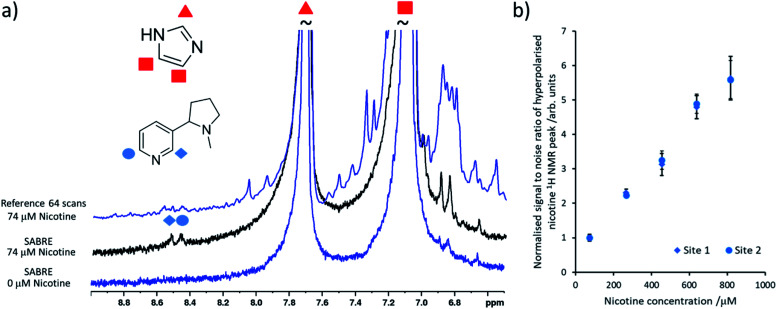
Quantitative low concentration detection of nicotine. (a) Partial single scan ^1^H NMR spectra recorded at 9.4 T and 298 K after a sample containing [IrCl(COD)(IMes)] (2 mM) and imidazole (15 equiv.) and the indicated nicotine concentration in 0.6 mL methanol-d_4_ was shaken with 3 bar *p*H_2_ for 10 seconds at 6.5 mT. The corresponding reference spectrum (not to scale) is shown above. (b) The hyperpolarised ^1^H NMR signal to noise ratios for the inequivalent *ortho* sites of free nicotine increases as its concentration is increased. These are calculated by taking the largest signal intensity value between *δ* = 8.53–8.49 and 8.42–8.48 ppm for nicotine site 1 and 2 respectively and dividing by spectral noise between *δ* = −1 and −5 ppm (see ESI, Section S1.4† for more details). Each data point is the average of three repeat measurements, the error bar represents the average of these.

It is important to select a coligand that does not dilute significant polarisation away from the analyte of interest and it must ideally contain ^1^H NMR signals that do not overlap with the analyte of interest. Here, the readily available coligand imidazole is employed as it is known to form stable SABRE complexes^21,38^ and shows only modest ^1^H NMR signal enhancements (50–100 fold,^39,40^ although other studies have increased this by deuteration of the catalyst and other mixed ligand approaches^38^). Samples were prepared based on previously reported concentrations^26^ that contained [IrCl(COD)(IMes)] (2 mM) and the coligand imidazole (15 equiv.) activated overnight with H_2_ (3 bar) in methanol-d_4_ (0.6 mL). When nicotine concentrations as low as 74 μM were added to this sample and *p*H_2_ shaking was performed, hyperpolarised nicotine ^1^H NMR signals for the two *ortho* sites could be discerned (the *meta* and *para* sites overlap with those of the imidazole coligand) (Fig. 2a). Additions of further amounts of nicotine, followed by fresh *p*H_2_ shaking after each addition, yields a linear relationship between nicotine concentration and its hyperpolarised ^1^H NMR signal intensity at these concentrations (Fig. 2b). This relationship breaks down when [Ir(H)_2_(IMes)(imidazole)_3_]Cl is no longer in excess compared to [Ir(H)_2_(NHC)(imidazole)_2_(nicotine)]Cl (see ESI, Fig. S13†).^24,26^ Nicotine concentrations of 34 μM or lower could not be detected in these single scan ^1^H NMR spectra. Others have reported the detection of *ca.* 1–2 μM amounts of N-heterocycles in single scan SABRE hyperpolarised ^1^H NMR measurements that utilise a 1-methyl-1,2,3-triazole coligand.^24,26^ Our inability to detect these concentrations is likely linked with modest polarisation transfer to the imidazole coligand, which should be reduced or prevented to increase the nicotine detection limit, which is estimated to be *ca.* 50 μM using this system.

**Fig. 3:**
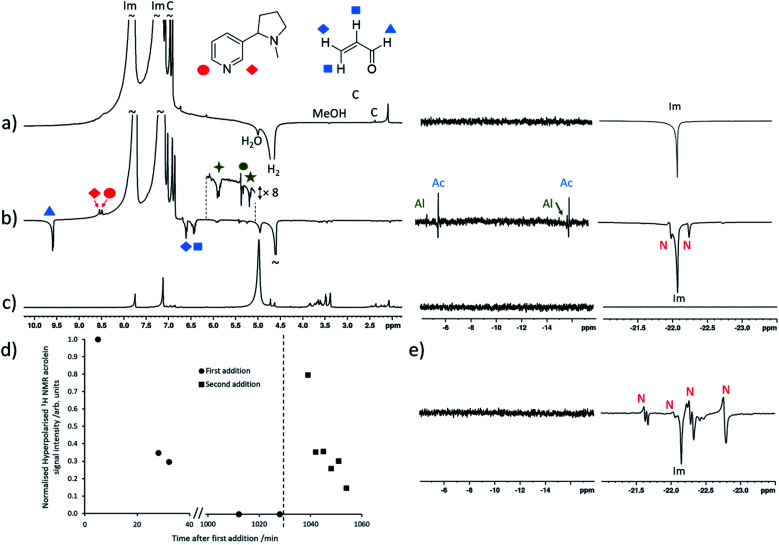
SABRE hyperpolarisation of electronic cigarette aerosol solutions. Partial single scan ^1^H NMR spectra recorded at 9.4 T and 298 K after a sample containing (a) [IrCl(COD)(IMes)] (5 mM) and imidazole (15 equiv.) in 0.5 mL methanol-d_4_ was shaken with 3 bar *p*H_2_ for 10 seconds at 6.5 mT. (b) SABRE-hyperpolarised ^1^H NMR spectrum after 50 μL electronic cigarette aerosol solution was added to the sample from (a) and *p*H_2_ shaking was repeated (time after addition = 5 min). (c) Thermally polarised ^1^H NMR spectrum of solution used in (b) (time after addition = 10 min). (d) Time course of hyperpolarised acrolein signals; the vertical dashed line indicates the point at which a further portion of electronic cigarette aerosol solution was added. (e) Partial single scan ^1^H NMR spectra recorded at 9.4 T and 298 K after the solution from (c) was doped with *ca.* 2.5 mM nicotine. Note that resonances labelled ‘Im’, ‘N’, or ‘C’ correspond to imidazole coligand and [Ir(H)_2_(IMes)(imidazole)_3_]Cl, [Ir(H)_2_(IMes)(imidazole)_2_(nicotine)]Cl (that can exist as two geometric isomers), and the IMes ligand of the SABRE catalyst respectively. Those signals marked by the green symbols are consistent with 1,4-pentadien-3-ol or related molecules. ‘Ac’ and ‘Al’ indicates signals likely arising from iridium-acrolein and 1,4-pentadien-3-ol bound adducts.

These measurements are next extended to the detection of nicotine, and other molecules, in electronic cigarette aerosol, for which our current detection limit is sufficient for analysis. 50 μL of the samples Ecig and Ecig^diluent^ were hyperpolarised by addition to a solution of [IrCl(COD)(IMes)] (5 mM) and imidazole (15 equiv.) which had been preactivated with H_2_ (3 bar) in methanol-d_4_ (0.5 mL). Shaking with *p*H_2_ produced hyperpolarised ^1^H NMR signals for nicotine in addition to a range of other hyperpolarised signals (Fig. 3b). Enhanced signals at *δ* = 9.56, 6.58 and 6.41 ppm correspond to acrolein which are enhanced by *ca.* 150-fold. We are unaware of any examples of aldehydes hyperpolarised using SABRE to date.^9^ A set of hyperpolarised signals at *δ* = 5.87, 5.36, 5.22 and 4.92 ppm are also observed immediately after the addition of electronic cigarette aerosol solution (Fig. 3b). 2D NMR characterisation of a reference sample of electronic cigarette aerosol solution confirm that these resonances belong to the same molecule (see ESI, Fig. S7†). A structure for this molecule cannot be assigned definitively, although its chemical shift values are consistent with 1,4-pentadien-3-ol and related molecules.^41^

Reversible coordination of both aldehyde and alcohol to the SABRE catalyst must be responsible for the observation of these SABRE-enhanced NMR signals. When the hydride region of their SABRE-hyperpolarised spectra are examined more closely, enhanced signals for species at *δ* = −5.85, −16.28 ppm and *δ* = −4.92, −16.14 ppm are observed in addition to those of [Ir(H)_2_(IMes)(imidazole)_3_]Cl and [Ir(H)_2_(IMes)(imidazole)_2_(nicotine)]Cl (Fig. 3b). Unfortunately, these species are of insufficient concentration or lifetime to allow their characterisation using 2D NMR spectroscopy. Nevertheless, the more dominant of these, at *δ* = −5.85, −16.28 ppm, is expected to correspond to a complex of the form [Ir(H)_2_(IMes)(imidazole)_2_(acrolein)]Cl which is responsible for the SABRE effect of the free aldehyde. It is anticipated that substrate coordination through the vinyl group is more likely and is supported by these hydride chemical shift values, which are not consistent with the *ca. δ* = −28 to −30 ppm range expected for iridium(iii) hydrides located *trans* to oxygen.^42–46^

Interestingly, when the shaking process is repeated several times following addition of fresh *p*H_2_, these enhanced acrolein and hydride signals decrease in intensity until they are no longer visible (Fig. 3d), while signals of nicotine remain enhanced with similar signal intensity. When a fresh 50 μL addition of the electronic cigarette aerosol solution is made, enhanced ^1^H NMR signals for acrolein, and those we speculate could belong to 1,4-pentadien-3-ol, and the associated hydride signals of their iridium-bound adducts, reappear before decreasing upon fresh *p*H_2_ shaking. Control measurements confirm that this behaviour is not due to evaporation of these analytes by sample degassing or replacement of *p*H_2_. Rather, this is indicative of a chemical reaction in which the analyte concentration decreases throughout the process. For example, many-scan thermal ^1^H NMR measurements on this solution after the reaction has occurred show that no acrolein signals remain, despite reference measurements of equivalent concentrations of electronic cigarette aerosol solution confirming its presence (see ESI, Fig. S14†). These observations can be explained by the hydrogenation of these analytes by the SABRE catalyst,^47,48^ although ^1^H NMR signals for any additional organic reaction products are not clearly discerned in either SABRE or thermally polarised measurements of this complex mixture. Related hydrogenation of a vinylsulfoxide using this precatalyst has been reported and similar reactivity of unsaturated aldehydes and alcohols is not unexpected.^49^

This decomposition makes quantification of these analytes challenging as their concentration changes during the measurements. Nevertheless, the diagnostic potential for rapid mixture analysis is highlighted by comparison of these results with those from Ecig^diluent^ that do not contain nicotine. When these experiments are repeated using Ecig^diluent^ aerosol solutions, the two samples are clearly distinguished based on the appearance of their SABRE-hyperpolarised ^1^H NMR spectra, which do not show any peaks for hyperpolarised nicotine. In this mixture, acrolein and the signals tentatively attributed to 1,4-pentadien-3-ol display similar enhanced ^1^H NMR signals (see ESI, Fig. S15†).

**Fig. 4:**
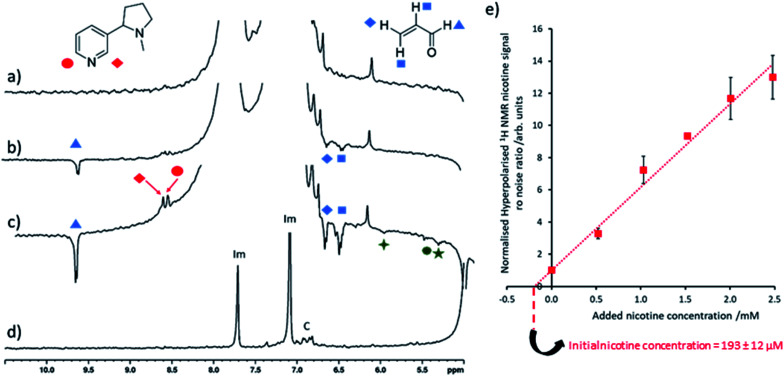
Quantitative low concentration nicotine detection using SABRE hyperpolarised ^1^H NMR (a) partial single scan ^1^H NMR spectra recorded at 9.4 T and 298 K after a sample containing [IrCl(COD)(IMes)] (2 mM) and imidazole (15 equiv.) in 0.6 mL methanol-d_4_ was shaken with 3 bar *p*H_2_ for 10 seconds at 6.5 mT. Analogous SABRE hyperpolarised ^1^H NMR spectrum after addition of (b) 1 μL and (c) 3 μL electronic cigarette aerosol solution to the sample used in (a). (d) Thermally polarised ^1^H NMR spectrum of the solution used in (c). Note that resonances labelled ‘Im’ and ‘C’ correspond to imidazole coligand and the IMes ligand of the SABRE catalyst respectively. Those signals marked by the green symbols are consistent with 1,4-pentadien-3-ol or related molecules. (e) The hyperpolarised ^1^H NMR signal to noise ratio of the nicotine *ortho* signals increase after known nicotine concentrations were added to the sample used in (c) and were used to calculate initial nicotine concentration from the line of best fit *x* axis intercept. Signal to noise ratios are calculated by taking the largest signal intensity value between *δ* = 8.53–8.49 and 8.42–8.48 ppm for nicotine site 1 and 2 respectively and dividing by spectral noise between *δ* = −1 and −5 ppm (see ESI, Section S1.4† for more details). Each data point is the average of three repeat measurements, the error bar represents the average of these. The error value on the initial nicotine concentration has been calculated by propagating the average 5% experimental error on SABRE experiments with the 2% error of the regression fitting.

The nicotine concentrations detected in electronic cigarette aerosol solutions using the SABRE-hyperpolarised NMR measurements discussed so far (Fig. 3) are estimated to be on the order of 2–3 mM which are still sufficient for detection using thermally polarised ^1^H NMR, with only modest time investment required for sufficient signal averaging. Therefore, these measurements are extended to detect much lower nicotine concentrations in Ecig aerosol for which thermally polarised ^1^H NMR may involve prohibitively long acquisition times. For example, hyperpolarised nicotine ^1^H NMR signals could be observed when 3 μL of the nicotine-containing Ecig aerosol solution was added to a preactivated sample of [IrCl(COD)(IMes)] (2 mM), imidazole (15 equiv.) and *p*H_2_ (3 bar) in methanol-d_4_ (0.6 mL) and SABRE experiments performed (Fig. 4c). Addition of further portions of known nicotine amounts can be used to determine the unknown concentration of nicotine as previously described (Fig. 4e). This yields a nicotine concentration of 193 ± 12 μM. Signal averaged thermally polarised ^1^H NMR spectra can be used to estimate a nicotine concentration by comparison of the integral intensity to those of the imidazole signals which correspond to a known concentration. This yields a nicotine concentration of *ca.* 187 μM which is consistent with SABRE-hyperpolarised data. These measurements contain an average reproducibility error of *ca.* 5%, which is sufficient for agreement between concentration values determined from hyperpolarised and thermally-polarised NMR measurements. The accuracy of the determined concentrations can likely be increased by improved experiment design, such as automated *p*H_2_ bubbling apparatus and/or flow systems that might control the polarisation transfer field more precisely.^8,50,51^ Smaller incremental increases of nicotine concentration during spiking may also improve accuracy as reproducibility errors increase at larger concentrations. Nevertheless, these SABRE-hyperpolarised NMR spectra involve a total measurement time of *ca.* 1 minute and can be performed by an experienced researcher in around 45 minutes (signal to noise ratio for nicotine of >35). This provides a time advantage compared to Boltzmann-polarised NMR, for which nicotine signals of a reference sample of equivalent concentration can be discerned with a signal to noise ratio of *ca.* 9 after 70 minutes of signal averaging. This time saving will become more pronounced when molecules are detected at even lower concentrations, for which the detection limit of our setup must be lowered.

## Conclusions

The SABRE-hyperpolarised NMR approach presented here can clearly make low concentration molecules such as nicotine and acrolein visible to single scan ^1^H NMR. The enhanced ^1^H NMR signals of acrolein are novel as, to the best of our knowledge, SABRE has not yet been used to enhance NMR signals of aldehydes.^9^ We demonstrate how SABRE can be used to enhance chemical analysis of complex mixtures by NMR and work towards a real-world example such as the analysis of electronic cigarette products. In the future, SABRE-hyperpolarised NMR may become a viable complementary method for chemical analysis in addition to established thermally polarised NMR or MS methods, the latter of which can be more sensitive. An average tobacco rod contains 10–14 mg nicotine^52^ and the electronic cigarette fluids sold for consumer use contain typical nicotine concentrations of 18 mg mL^−1^. In electronic cigarette aerosols, nicotine concentration is in the mg region.^53^ Therefore, the current detection range of single scan SABRE-hyperpolarised ^1^H NMR is more than adequate to observe nicotine and other molecules in tobacco products or electronic cigarette aerosols.

In the future these results can be improved by lowering the analyte detection limit. This could be achieved by limitation of the modest (<200-fold) ^1^H NMR polarisation of the imidazole coligand by approaches such as deuteration,^10^ or even selection of an alternative coligand,^24,26^ which could also reduce chemical shift overlap. A lower analyte detection limit will also improve the time savings that SABRE-hyperpolarised NMR can provide compared to other NMR methods. Closely related hyperpolarisation methods, such as relayed polarisation transfer (SABRE-Relay) has recently been developed to enhance the NMR signals of OH-containing molecules that do not ligate to the metal SABRE catalyst.^54^ These measurements could be extended to low concentration detection of other OH-containing molecules present in these cigarette aerosol solutions in the future. We demonstrate how SABRE-hyperpolarised NMR could be used in a real-world application to aid molecular profiling of electronic cigarette aerosols which may be of great use in rapid distinctions between different aerosol solutions.

## Data availability statement

Data for this paper, including 1D and 2D experimental NMR data of the article are available at IDA repository at http://urn.fi/urn:nbn:fi:att:04e3bfee-0fd0-43c5-8bbb-59b7da7ad9a8.

## Author contributions

BJT: conceptualisation, investigation, validation, visualisation, writing – original draft, review and editing; SK: conceptualisation, investigation, validation, writing –review and editing; SP: providing aerosol solutions, writing –review and editing; JH: providing aerosol solutions; PL: funding acquisition, supervision; VVZ: funding acquisition, resources, synthesis of [IrCl(COD)(IMes)] precatalyst, supervision, writing – review and editing; VVT: conceptualisation, funding acquisition, resources, supervision, writing – review and editing.

## Conflicts of interest

There are no conflicts to declare.

## Supplementary Material

RA-012-D1RA07376A-s001
