# Supplementary material for: Hyperpolarised NMR to aid molecular profiling of electronic cigarette aerosols
Source: RSC Adv. 2022 Jan 10;12(3):1479–85. doi: 10.1039/d1ra07376a (PMC8979170; doi:10.1039/d1ra07376a)
Supplement: RA-012-D1RA07376A-s001 [file RA-012-D1RA07376A-s001.pdf]

Electronic Supplementary Material (ESI) for RSC Advances.
This journal is © The Royal Society of Chemistry 2021

Supporting Information

Hyperpolarised NMR to aid molecular profiling of electronic cigarette aerosols

Ben. J. Tickner[a†], Sanna Komulainen[a†], Sanna Palosaari[b,c], Janne Heikkinen,[b] Petri Lehenkari[b,c,d], Vladimir V. Zhivonitko[a]* and Ville-Veikko Telkki[a]*

[a] NMR Research Unit, Faculty of Science, University of Oulu, P.O. Box 3000, 90014 Oulu, Finland.

[b] Cancer and Translational Medicine Research Unit, Faculty of Medicine, University of Oulu, Finland, 90014

[c] Medical Research Center Oulu, Faculty of Medicine, University of Oulu and Oulu University Hospital, Finland, 90014.

[d] Division of Orthopedic Surgery, Oulu University Hospital, Finland, 90220

[†] These authors contributed equally to this work.

* Corresponding authors: vladimir.zhivonitko@oulu.fi and ville-veikko.telkki@oulu.fi

Table of Contents

S1. Experimental

S1.1 General Remarks

S1.2 Preparation of Ecig and Ecig^{diluent} samples

S1.3 Formation and utilisation of parahydrogen

S1.4 SABRE hyperpolarisation measurements

S2. Analysis of electronic cigarette aerosol solutions using thermally polarised NMR

S2.1 Thermally polarised NMR characterisation of Ecig

S2.2 Thermally polarised NMR characterisation of Ecig^{diluent}

S3. SABRE hyperpolarisation of nicotine

S3.1 2D NMR characterisation of nicotine

S3.2 2D NMR characterisation of $[Ir(H)_2(IMes)(nicotine)_3]Cl$

S3.3 SABRE hyperpolarisation of nicotine

S3.4 Linearity of hyperpolarised nicotine ^{1}H NMR signals

S4. Analysis of electronic cigarette aerosol solutions using SABRE hyperpolarised NMR

S4.1 SABRE Hyperpolarised NMR of Ecig

S4.2 SABRE Hyperpolarised NMR of Ecig^{diluent}

S5. References

S1. Experimental

S1.1 General Remarks

All NMR measurements were carried out on a 400 MHz Bruker Avance III spectrometer using solutions at room temperature (298 K). ^{1}H (400 MHz) and ^{13}C (100.6 MHz) NMR spectra were recorded with an internal deuterium lock. Chemical shifts are quoted as parts per million and referenced to the solvent. ^{13}C NMR spectra were recorded with broadband proton decoupling. Absolute values of coupling constants (J) are quoted in Hertz and rounded to the nearest 0.5 Hz.

All commercial compounds were purchased from Sigma-Aldrich and used as supplied unless otherwise stated. [IrCl(COD)(IMes)] (where IMes = 1,3-bis(2,4,6-trimethyl-phenyl)imidazole-2-ylidene and COD = cis,cis-1,5-cyclooctadiene) was synthesized according to literature procedures.[1] (-)-nicotine was used for reference measurements and spiking samples with known amounts of nicotine.

Samples were prepared containing either 2 or 0.8 mg [IrCl(COD)(IMes)] precatalyst and the indicated substrate (nicotine) and/or coligand in 0.5 or 0.6 mL of methanol-d_4 in a 5 mm NMR tube that was fitted with a quick pressure valve. The resulting solutions were degassed by three freeze-pump-thaw cycles before the addition of 3-bar pH_2. In cases where nicotine, or electronic cigarette aerosol solutions, were added to samples containing SABRE active catalyst the solution was frozen in liquid nitrogen before the valve was removed and the addition made. The solution was then degassed using a vacuum pump to remove air and the solution melted.

S1.2 Preparation of Ecig and Ecig^{diluent} samples

Two samples were prepared. Ecig contained aerosol of electronic cigarette fluid containing 18 mg/mL nicotine mixed with 50:50 VG18 and PG18 NicBase where VG and PG are vegetable glycerine and propylene glycol respectively (Chemnovatic, Lublin, Poland). Ecig^{diluent} consisted only of aerosol of the electronic cigarette carrier fluid 50VG:50PG (Lubrisolve, Somerton, UK). Both liquids were heated with eGO AIO vaporizer (Joyetech Electronics Co., Ltd., Shenzhen, China) for 12 x 10 second pulses and the vapour was led through 2 mL methanol-d_4 using a vacuum system. A photograph of the apparatus used to prepare these aerosol solutions is shown in Figure S1.

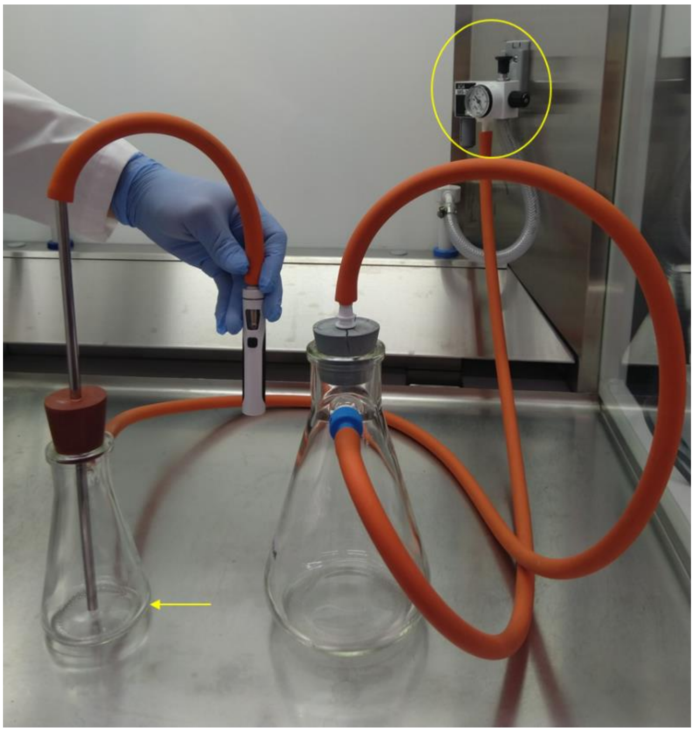

Figure S1: The vacuum system for preparation of electronic cigarette aerosol solutions. The sample collection flask is indicated by an arrow and the vacuum measurement instrumentation by a circle.

S1.3 Formation and utilisation of parahydrogen

Hydrogen gas was produced from the electrolysis of water using a desktop hydrogen generator (F-DGSi, Evry, France). This was used directly to make parahydrogen (pH_2) using a BPHG 90 parahydrogen generator (Bruker) which passes the hydrogen gas over a spin-exchange catalyst at low temperature. The generator operates at *ca* 38 K and produces a constant flow of pH_2 with *ca* 92% purity. Parahydrogen (3 bar) was added to NMR tubes containing a quick pressure valve using a home-built system shown in Figure S2. This set-up contains 3 valves that allow the system to be opened to A) the NMR tube B) parahydrogen and C) a vacuum pump. This system was used to degas the sample (see Section S1.1).

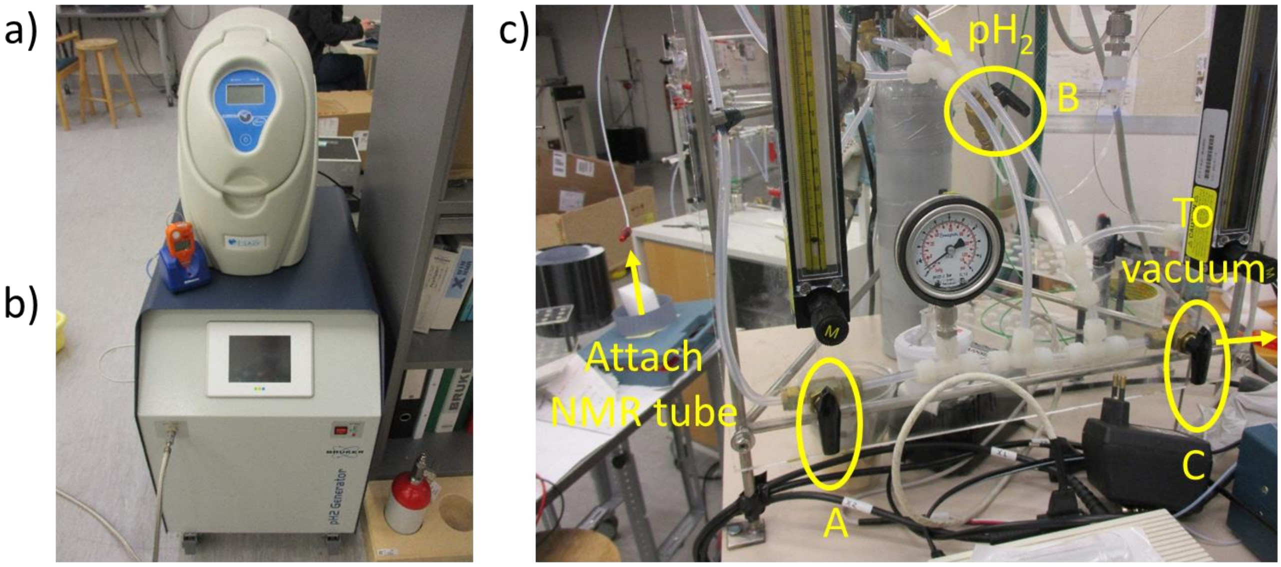

Figure S2: Picture of a) hydrogen generator b) parahydrogen generator and c) home-built device for addition of parahydrogen to NMR tubes in which A, B and C refer to the valves that allow opening of the system to the NMR tube, parahydrogen line or vacuum pump respectively.

Samples were left to activate for a period of several hours (typically around 16 hours overnight). Activation is usually accompanied by a change in colour from orange to pale orange. Catalyst activation is indicated in ^1H NMR spectra by the formation of a peak corresponding to $[Ir(H)_2(IMes)(L)_3]Cl$ between $\delta = -21$ and -24 ppm. As catalyst activation proceeds, ^1H NMR signals for the COD ligand at δ = 3 and 4 ppm eventually disappear and a signal for cyclooctane at *ca* δ = 1.6 ppm becomes visible.[2–4] When solutions involving catalyst loadings of 5 mM are used, catalyst activation to form $[Ir(H)_2(IMes)(imidazole)_3]Cl$ generally gives rise to solutions containing a single hydride ^1H NMR peak in the region at $\delta = -22.34$ ppm.[5] We note that under dilute conditions (2 mM catalyst loadings) activation of $[Ir(H)_2(IMes)(imidazole)_3]Cl$ proceeds less cleanly. In these solutions, other hydride ^1H NMR signals in addition to those of $[Ir(H)_2(IMes)(imidazole)_3]Cl$ are observed. We attribute this to formation of solvent bound adducts such as $[Ir(H)_2(IMes)(imidazole)_2(ODCD_3)]Cl$ which are likely responsible for a signal sometimes observed at $\delta = -28.87$ ppm or other impurity bound adducts which appear more likely to form at higher solvent:catalyst ratios. These side products appear to reduce SABRE efficiency[6,7] of imidazole and therefore in the experiments detailed here we discard solutions that contain significant amounts of these impurities.

S1.4 SABRE hyperpolarisation measurements

The shake and drop method was employed for recording hyperpolarised NMR spectra. This involves filling NMR tubes with fresh pH_2 (3 bar) as described in Section S1.2 before shaking them vigorously for 10 seconds in a 6.5 mT magnetic field.[2] We find that the stray field of our shielded 9.4 T magnet is no larger than 2 mT, therefore we use an electromagnetic coil powered by a Blanko PS-3005 0-30V 0-5A switching power supply to provide the necessary magnetic fields for SABRE polarisation transfer. The current and voltage of the power supply can be altered to achieve a magnetic field inside the coil of *ca* 6.5 mT. Magnetic fields were measured using a Hirst GM04 Gaussmeter and we estimate that the sample experiences a magnetic field of 6.5 ± 1.0 mT during the manual shaking process. This set up is shown in Figure S3 and is placed as close to the NMR spectrometer as possible to reduce transfer time after the 10 second shaking period. The sample is inserted into the spectrometer as rapidly as possible, this is facilitated by pre-emptively turning off the lift function on the spectrometer. ^{1}H NMR pulse sequences are modified to include an autosuspend function such that radiofrequency excitation occurs immediately upon sample insertion.

^{1}H NMR signal enhancements were calculated by dividing the hyperpolarised signal integral intensity by their corresponding integrals in a thermally polarised spectrum. It is essential that both spectra are recorded and processed using the same spectral acquisition parameters. Unless otherwise stated, multiple shake and drop measurements were undertaken and average ^{1}H NMR signal enhancement values quoted. Signal to noise ratios were calculated using the topspin 'sino' command which takes the largest intensity value of a signal within a given frequency window and divides it by spectral noise within a second frequency window of the same spectrum. The same frequency windows were used throughout this work: for the inequivalent nicotine *ortho* signals at δ = 8.52 and 8.46 ppm, the maximal signal intensity between δ = 8.53-8.49 and 8.42-8.48 ppm respectively were used with the region between $\delta = -1$ and -5 ppm (which contains no signals) used for the calculation of spectral noise.

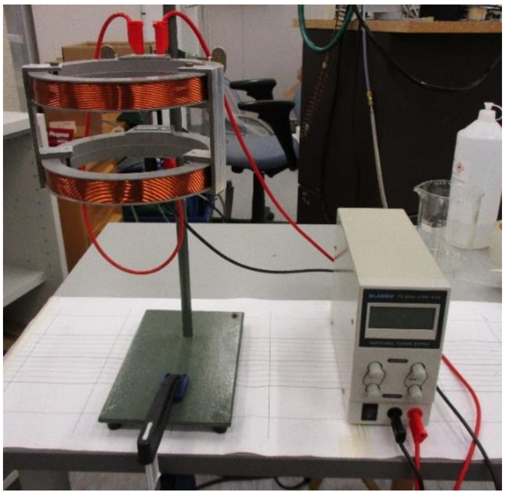

Figure S3: Picture of electromagnetic coil and power supply used to generate the 6.5 mT fields necessary for efficient SABRE polarisation transfer.

S2. Analysis of electronic cigarette aerosol solutions using thermally polarised NMR

S2.1 Thermally polarised NMR characterisation of Ecig

Thermally polarised 1D and 2D NMR (COSY, NOESY, HMQC) were used to characterise some of the molecules present within the electronic cigarette aerosol solutions. For these experiments, samples containing electronic cigarette aerosol (100 µL) in methanol-*d*4 (0.5 mL) were used. NMR characterisation was performed at 9.4 T and 298 K. An example ^{1}H NMR and ^{1}H-^{1}H COSY spectrum is shown in Figure S4 and S5 respectively with NMR characterisation data presented in Table S1.

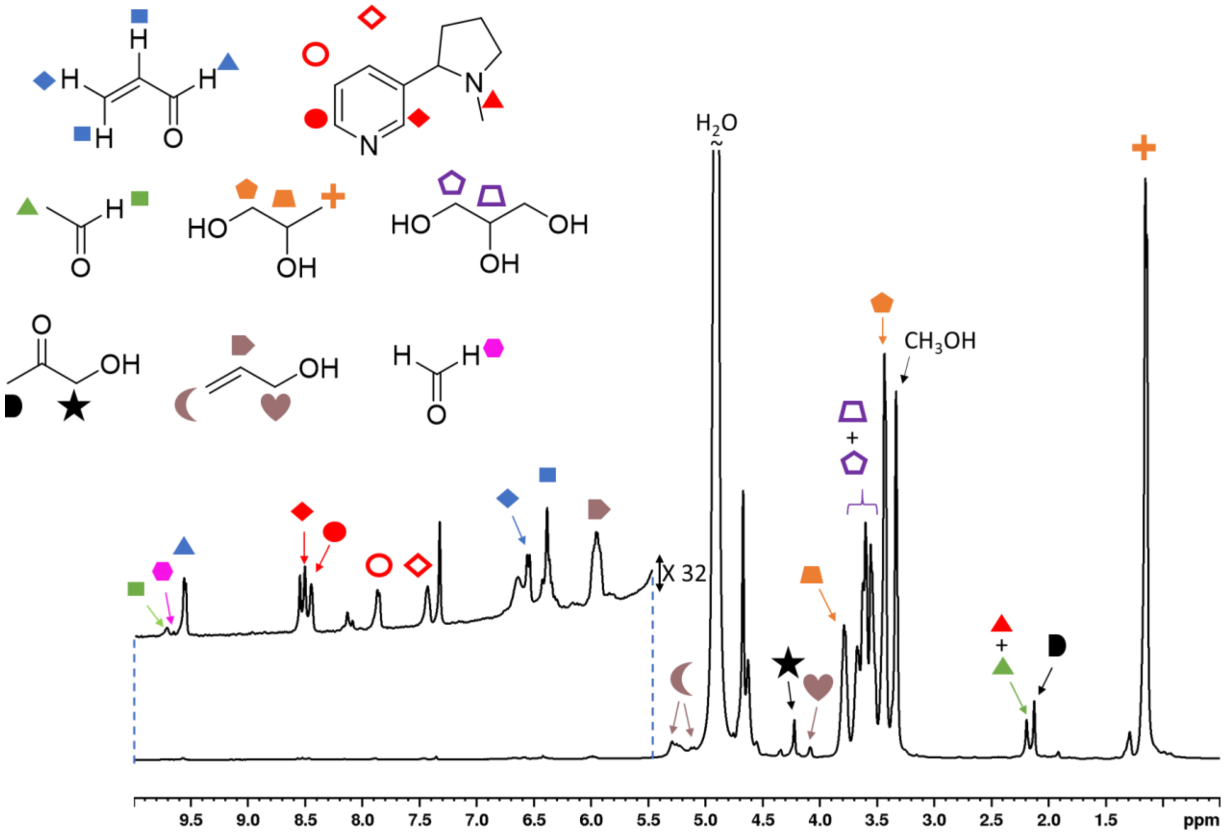

Figure S4: 512 scan ^{1}H NMR spectra recorded at 9.4 T and 298 K of a solution of electronic cigarette aerosol (100 µL) in methanol-d4 (0.5 mL).

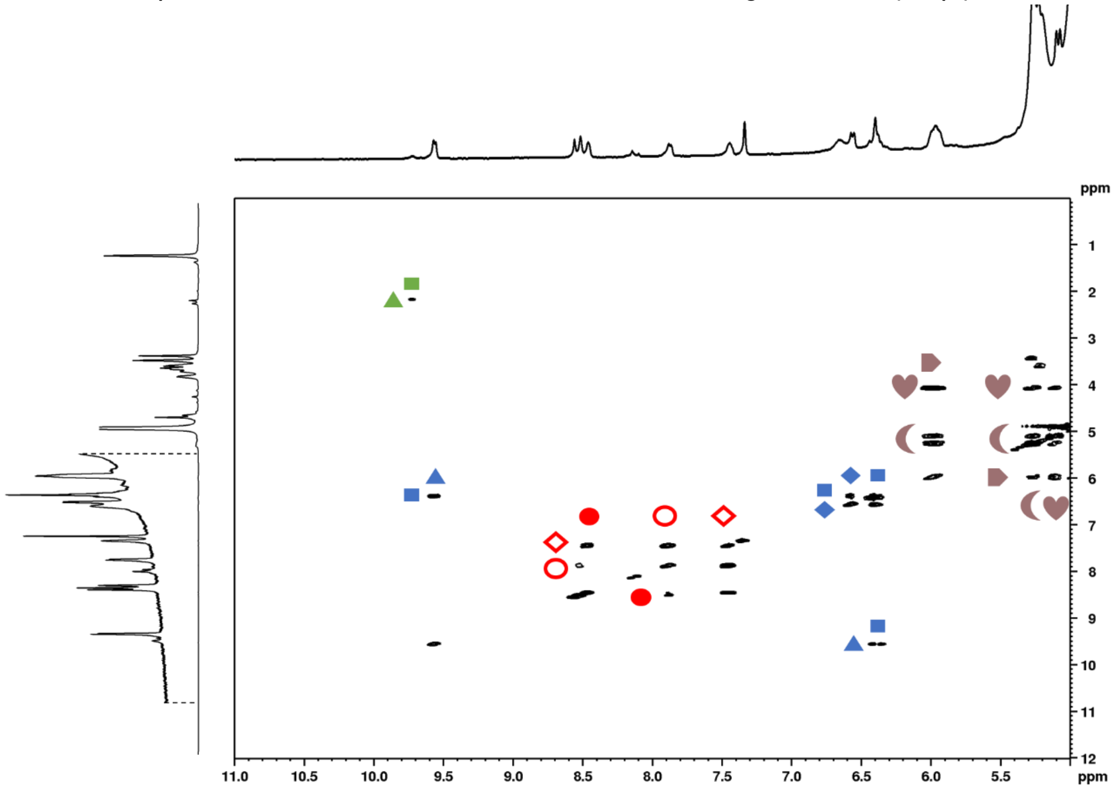

Figure S5: Partial ^{1}H-^{1}H COSY spectra recorded at 9.4 T and 298 K of a solution of electronic cigarette aerosol (100 µL) in methanol-d4 (0.5 mL).

S2.2 Thermally polarised NMR characterisation of Ecig^{diluent}

Thermally polarised 1D and 2D NMR (COSY, NOESY, HMQC) were used to characterise some of the molecules present within the electronic cigarette aerosol solutions. For these experiments, samples containing Ecig^{diluent} (100 µL) in methanol-d_4 (0.5 mL) were used. NMR characterisation was performed at 9.4 T and 298 K. An example ^{1}H NMR and ^{1}H-^{1}H COSY spectrum is shown in Figure S6 and S7 respectively.

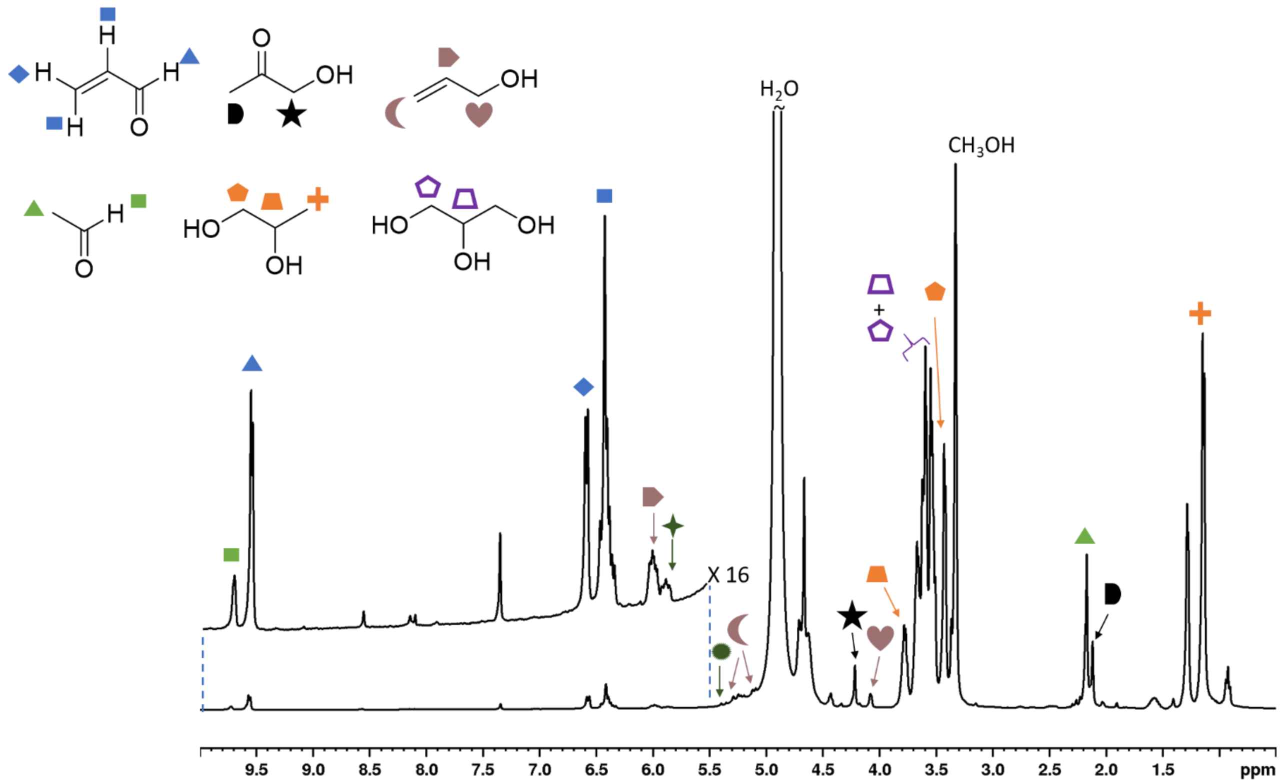

Figure S6: 512 scan ^{1}H NMR spectra recorded at 9.4 T and 298 K of a solution of nicotine-free electronic cigarette aerosol (100 µL) in methanol-d$_4$ (0.5 mL). Those signals marked by the green symbols are consistent with 1,4-pentadien-3-ol or related molecules.

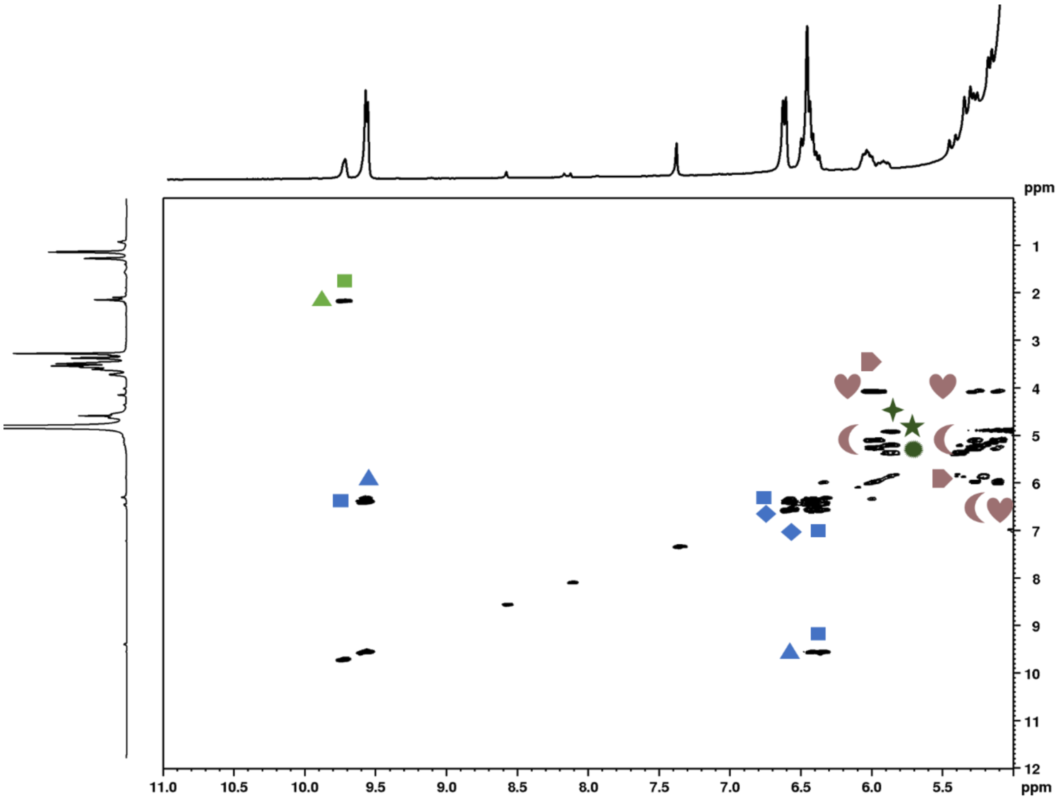

Figure S7: Partial ^{1}H-^{1}H COSY spectra recorded at 9.4 T and 298 K of a solution of Ecig^{diluent} aerosol (100 µL) in methanol-d$_4$ (0.5 mL). Those signals marked by the green symbols are consistent with 1,4-pentadien-3-ol or related molecules.

Table S1: NMR characterisation data for various molecules found to exist in the electronic cigarette aerosol mixtures.

Molecule		Resonance Position	^{1}H Chemical Shift	^{13}C Chemical Shift
Structure	Name			
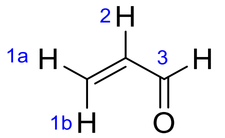	Acrolein	1a<br>1b<br>2<br>3	6.58 (d, *J* = 8.5 Hz)<br>6.42 (m) (overlap)<br>6.42 (m) (overlap)<br>9.57 (d, *J* = 6.5 Hz)	N/A<br><br>N/A<br>195.37
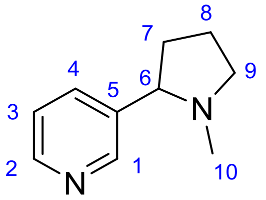	Nicotine	1<br>2<br>3<br>4<br>5-9<br>10	8.52<br>8.47<br>7.88 (d, *J* = 7.0 Hz)<br>7.46<br>N/A<br>2.19	N/A<br>N/A<br>N/A<br>N/A<br>N/A<br>N/A
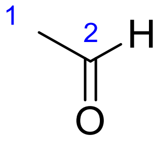	Acetaldehyde	1<br>2	2.19<br>9.73	29.37<br>204.78
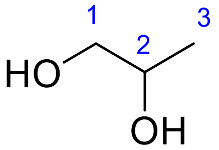	Propylene gylcol	1<br>2<br>3	3.43 (m)<br>3.78 (m)<br>1.15 (d, *J* = 5.0 Hz)	67.13<br>67.87<br>18.10
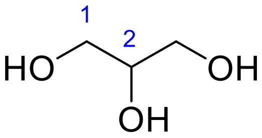	Glycerol	1<br>2	3.5-3.7 (m) (overlap)<br>3.5-3.7 (m) (overlap)	63.03<br>72.47
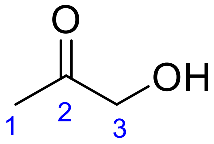	Hydroxyacetone	1<br>2<br>3	2.13<br>-<br>4.22	~ 24 (overlap)<br>208.94<br>67.72
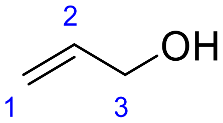	1-propenol	1<br>2<br>3	5.11 (d, *J* = 10 Hz), 5.27 (m)<br>5.98 (m)<br>4.08	113.61<br>N/A<br>N/A
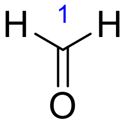	Formaldehyde	1	9.67	N/A
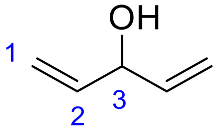	N/A<br>(consistent with 1,4-pentadien-3-ol)	1<br>2<br>3	5.22, 5.36<br>5.87<br>4.92	115.73<br>N/A<br>N/A
N/A	N/A	N/A<br>N/A<br>N/A	0.94 (t, *J* = 7 Hz)<br>1.59 (m)<br>4.43	N/A<br>29.38<br>99.62
N/A	N/A	N/A<br>N/A<br>N/A	1.29<br>4.71<br>N/A	21.1<br>95.28<br>101.98
N/A	N/A	N/A<br>N/A	4.67<br>N/A	90.1<br>52.9

S3. SABRE hyperpolarisation of nicotine

S3.1 2D NMR characterisation of nicotine

A solution containing nicotine (2.4 µL) in methanol-d_4 (0.6 mL) was characterised using 2D NMR spectroscopy (HMQC, COSY, NOESY) at 298 K at 9.4 T. The structure of nicotine is shown in Figure S8; full NMR characterisation data is given in Table S2 and is consistent with those presented elsewhere (see https://bmrb.io/metabolomics/mol_summary/show_data.php?id=bmse000105).

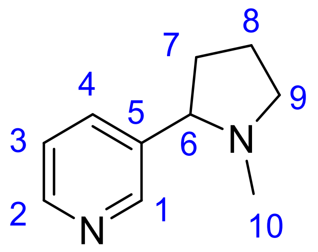

Figure S8: Structure of nicotine.

Table S2: NMR resonances of nicotine collected at 9.4 T and 298 K in methanol-d_4 with the resonance positions corresponding to those shown in Figure S8.

Resonance Position	^{1}H Chemical Shift /ppm	^{13}C Chemical Shift /ppm
1	8.52 (s)	148.49
2	8.46 (d, $^3J_{H2H1}$ = 4 Hz)	147.75
3	7.45 (m)	124.21
4	7.88 (d, $^3J_{H4H3}$ = 8 Hz)	136.09
5	-	138.71
6	3.22 (m)	68.75
7	1.81 (m), 2.29 (m)	34.51
8	1.93 (m), 2.00 (m)	21.98
9	2.39 (q, J = 9 Hz), 3.26 (m)	56.56
10	2.20 (s)	39.21

S3.2 2D NMR characterisation of $[Ir(H)_2(IMes)(nicotine)_3]Cl$

A solution containing [IrCl(COD)(IMes)] (5 mM) (where COD is *cis*,*cis*-1,5-cyclooctadiene and IMes is 1,3-bis(2,4,6-trimethylphenyl)imidazol-2-ylidene) and nicotine (2.4 µL, 5 equiv.) in methanol-d_4 (0.6 mL) was activated with H_2 (3 bar) overnight at room temperature. This solution was then characterised using 2D NMR spectroscopy (HMQC, COSY, NOESY) at 298 K at 9.4 T. The structure of $[Ir(H)_2(IMes)(nicotine)_3]Cl$ is shown in Figure S9; full NMR characterisation data is given in Table S3. Example COSY and HMQC spectra are presented in Figure S10 and S11 respectively. These data are consistent with similar values presented in Ref. [8]

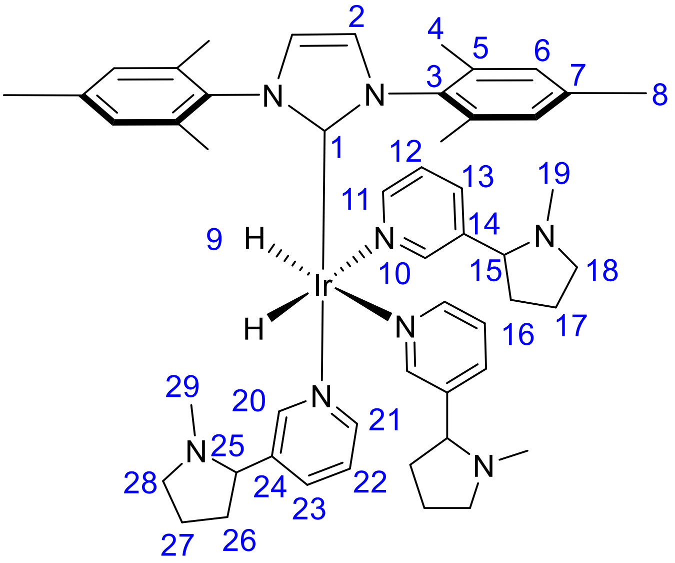

Figure S9: Structure of $[Ir(H)_2(IMes)(nicotine)_3]Cl$ determined from the NMR data shown in the Table S3.

Table S3: ^1H and ^{13}C NMR resonances of $[Ir(H)_2(IMes)(nicotine)_3]Cl$ collected at 9.4 T and 298 K in methanol-d_4 with the resonance positions corresponding to those shown in Figure S9.

Resonance Position	^1H Chemical Shift	^{13}C Chemical Shift
1	-	N/A
2	7.11	122.52
3	-	135.26
4	2.07	18.14
5	-	137.76
6	6.71	128.58
7	-	138.12
8	2.19	20.44
9	−22.67 d ($^2J_{HD}$ = 35 Hz)	-
10	8.36, 8.45 *	~152 (overlap)
11	8.32, 8.26 *	~152 (overlap)
12	7.12, 7.07 *	124.88
13	7.74	135.16
14	-	N/A
15	3.00	68.27
16	2.15	34.32
17	1.64	21.83
18	2.41, 3.25	56.34
19	2.01	39.44
20	7.62	153.23
21	8.18	154.96
22	6.98	124.63
23	7.61	135.56
24	-	N/A
25	2.74	67.81
26	1.19	34.41
27	1.84, 1.94	21.80
28	2.27, 3.22	56.14
29	1.78	38.92

*Two resonances are observed, these effects have been observed in other works and have been attributed to the inequivalence of the two nicotine ligands which is a result of nicotine chirality.[8,9]

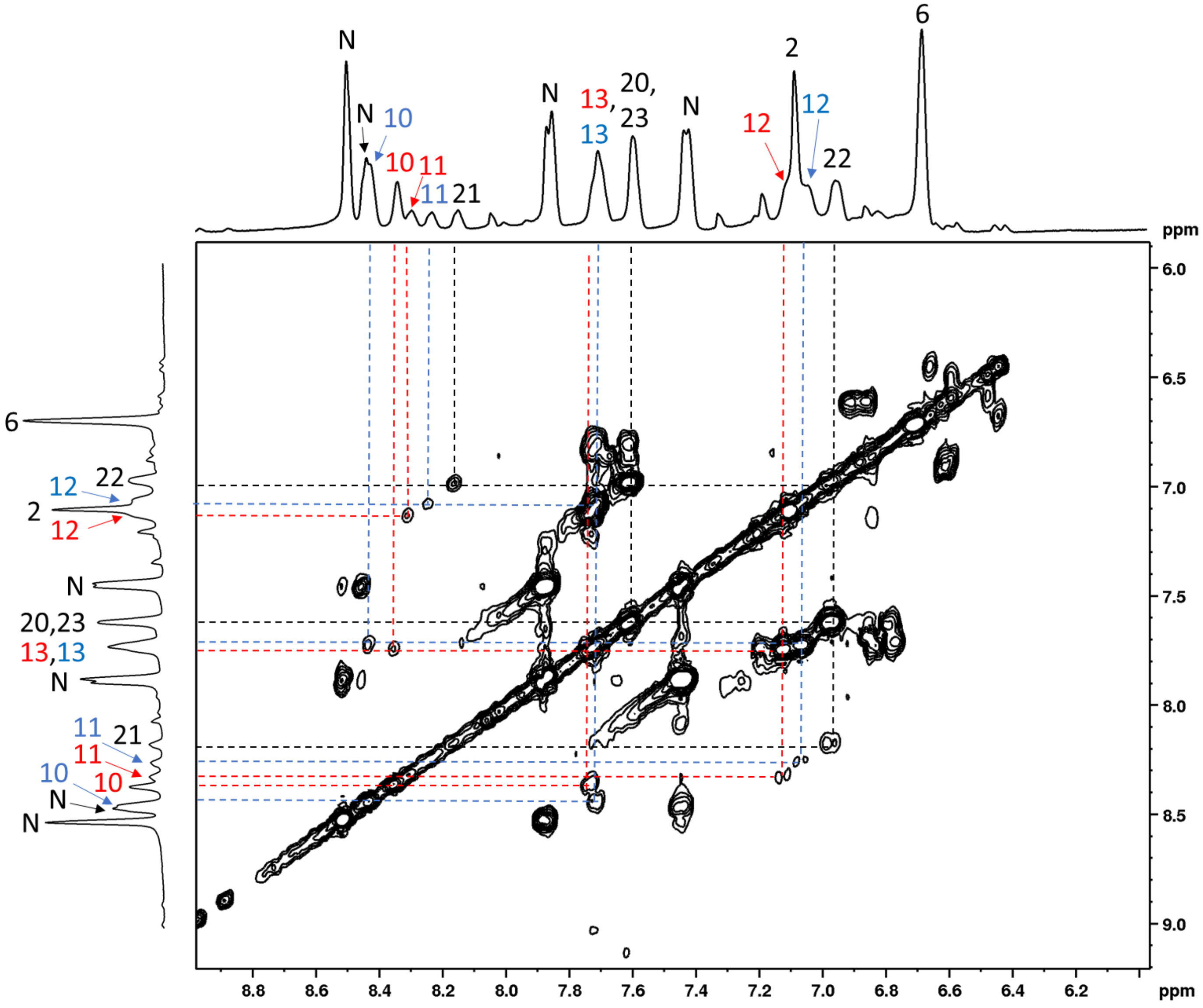

Figure S10: Partial ^{1}H COSY spectrum of $[Ir(H)_2(IMes)(nicotine)_3]Cl$ in methanol-d_4 at 298 K. Resonances labelled 'N' correspond to free nicotine. The other resonance labels correspond to those denoted in Figure S9. The resonance labels are colour coded red and blue and correspond to inequivalent nicotine ligands trans to hydride sites (i.e. signals in red arise from the same bound nicotine ligand trans to hydride, those in blue are from the other inequivalent nicotine ligand bound trans to hydride).

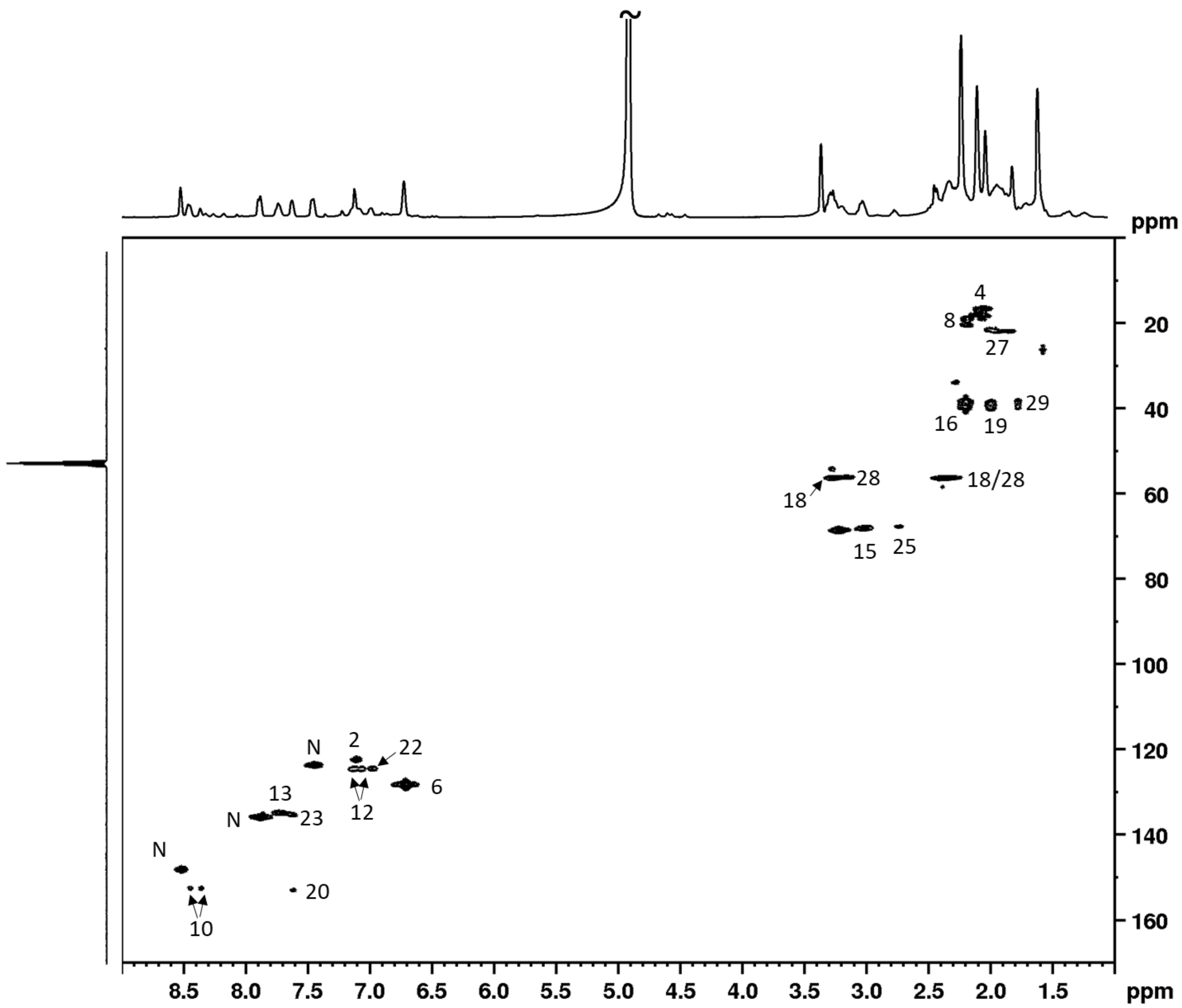

Figure S11: Partial ^{1}H-^{13}C HMQC, showing direct ^{1}H-^{13}C couplings, of [Ir(H)$_2$(IMes)(nicotine)$_3$]Cl in methanol-d_4 at 298 K. Resonances labelled 'N' correspond to free nicotine. The other resonance labels correspond to those denoted in Figure S9.

S3.3 SABRE hyperpolarisation of nicotine

A solution of $[Ir(H)_2(IMes)(nicotine)_3]Cl$ was shaken vigorously with pH_2 (3 bar) for 10 seconds at 6.5 mT before rapid sample insertion into a 9.4 T NMR spectrometer for collection of a single scan ^{1}H NMR spectrum. These spectra yield enhanced ^{1}H NMR signals at δ = 8.52, 8.46, 7.45 and 7.88 ppm corresponding to the two inequivalent *ortho*, *meta* and *para* resonances of the free nicotine pyridine ring respectively which are 66 ± 9, 65 ± 12, 107 ± 19 and 87 ± 17 times larger than those recorded under Boltzmann conditions respectively (Figure S12). None of the ^{1}H NMR signals of the nicotine pyrrolidine ring were hyperpolarised in these measurements. An enhanced hydride signal for $[Ir(H)_2(IMes)(nicotine)_3]Cl$ at δ = −22.67 ppm is also observed. Signals for nicotine located *trans* to hydrides within $[Ir(H)_2(IMes)(nicotine)_3]Cl$ are also enhanced with ^{1}H NMR signal gains of 65 ± 12, 78 ± 17, 41 ± 8 and 58 ± 13 -fold for the two *ortho*, *meta* and *para* sites respectively (Figure S12).

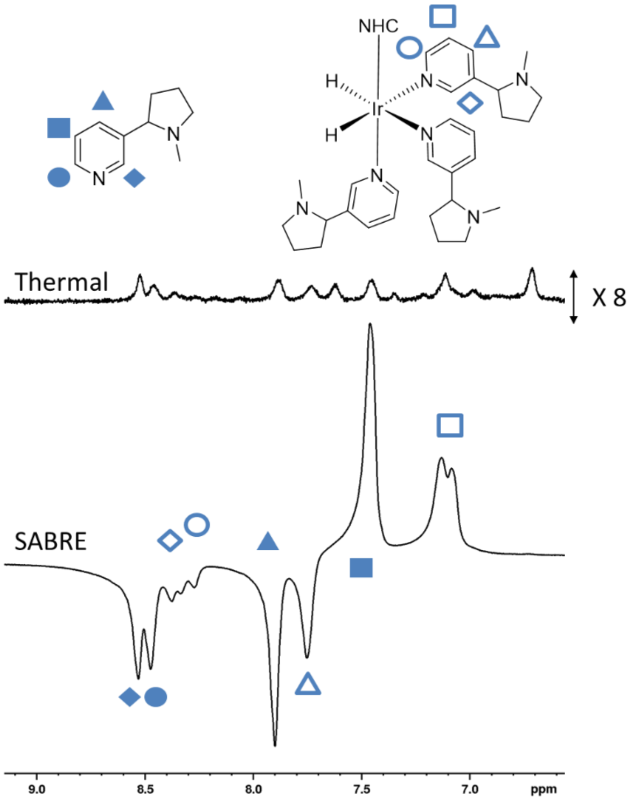

***Figure S12:** Hyperpolarisation of nicotine using SABRE. Partial single scan ^{1}H NMR spectra recorded at 9.4 T and 298 K after a sample containing [IrCl(COD)(IMes)] (5 mM) and nicotine (5 equiv.) in 0.6 mL methanol-d_4 were shaken with 3 bar pH_2 for 10 seconds at ca 6.5 mT (lower). The corresponding single scan thermal trace (expanded vertically by a factor of 8) is shown above.*

S3.4 Linearity of hyperpolarised nicotine ^{1}H NMR signals

A solution containing [IrCl(COD)(IMes)] (5 mM) (where COD is *cis*,*cis*-1,5-cyclooctadiene and IMes is 1,3-bis(2,4,6-trimethyl-phenyl)imidazol-2-ylidene) and imidazole (5 equiv.) in methanol-d_4 (0.6 mL) was activated with H_2 (3 bar) overnight at room temperature. Nicotine was added to this solution to give nicotine concentrations of between 400 µM and 13 mM (molar catalyst:nicotine ratio of between 1:0.08 and 1:2.68). When this solution was shaken with 3 bar pH_2 for 10 seconds at 65 G, enhanced ^{1}H NMR signals for nicotine were observed, but they did not increase linearly as a function of its concentration (Figure S13) as [Ir(H)$_2$(IMes)(imidazole)$_3$]Cl is no longer in excess compared to [Ir(H)$_2$(IMes)(imidazole)$_2$(nicotine)]Cl.[10,11]

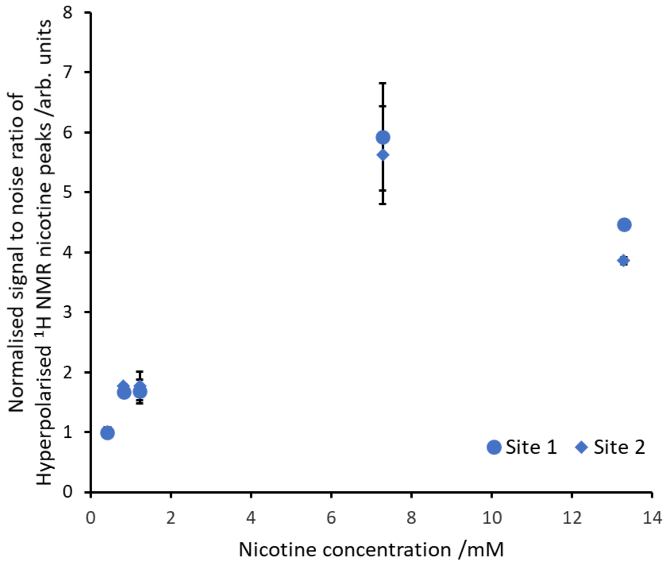

***Figure S13:** Normalised signal to noise ratios for hyperpolarised nicotine ^{1}H NMR signals as a function of nicotine concentration when it is shaken with 3 bar pH_2 for 10 seconds at 65 G in a sample containing [IrCl(COD)(IMes)] (5 mM) and imidazole (5 equiv.) in 0.6 mL methanol-d_4. Signal to noise ratios are calculated by taking the largest signal intensity value between δ = 8.53-8.49 and 8.42-8.48 ppm for nicotine site 1 and 2 respectively and dividing by spectral noise between δ = −1 and −5 ppm (see Supporting information, Section S1.4 for more details).*

S4. Analysis of electronic cigarette aerosol solutions

S4.1 SABRE Hyperpolarised NMR of Ecig

A solution containing [IrCl(COD)(IMes)] (5 mM) (where COD is *cis,cis*-1,5-cyclooctadiene and IMes is 1,3-bis(2,4,6-trimethyl-phenyl)imidazol-2-ylidene) and imidazole (15 equiv.) in methanol-d_4 (0.5 mL) was activated with H_2 (3 bar) for a few hours at room temperature. At this point 50 µL of the electronic cigarette aerosol solution was added before pH_2 shaking was performed (3 bar for 10 seconds at 6.5 mT). Later, a second 50 µL addition of the electronic cigarette aerosol solution was performed. After these experiments were performed, many scan reference ^{1}H NMR spectra were collected and compared to analogous measurements recorded on a sample containing the same electronic cigarette aerosol solution (100 µL) in methanol-d_4 (0.5 mL). A comparison between these spectra is shown in Figure S14 and shows that acrolein signals are no longer present in the solution containing the SABRE catalyst. More detailed 2D NMR characterisation of the electronic cigarette aerosol sample is presented in Section S2.

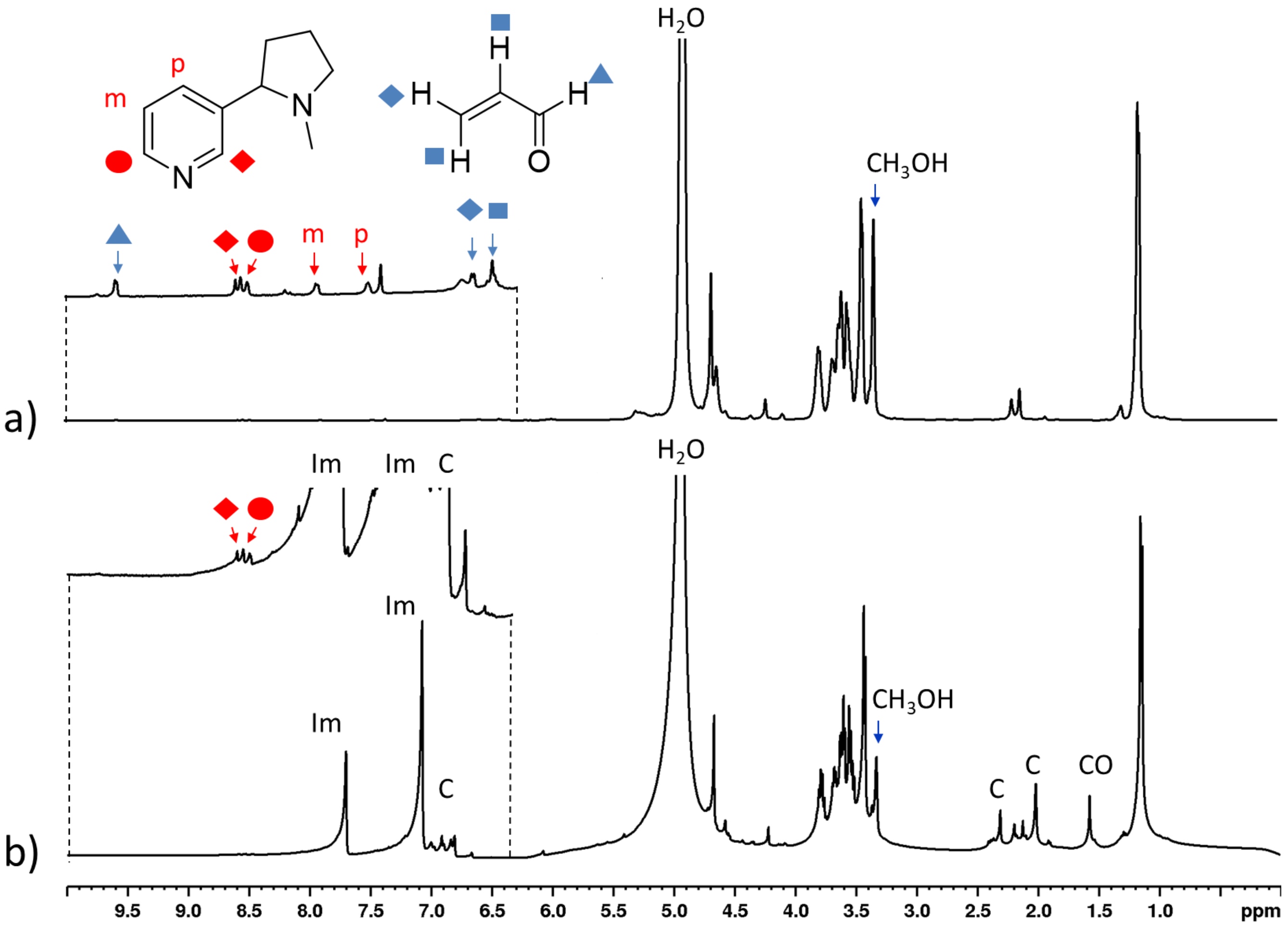

***Figure S14:** Thermally polarised ^{1}H NMR spectra recorded at 9.4 T and 298 K of a) 100 µL electronic cigarette aerosol in 0.5 mL methanol-d_4 and b) [IrCl(COD)(IMes)] (5 mM), imidazole (15 equiv.) and 100 µL electronic cigarette aerosol solution in 0.5 mL methanol-d_4 several hours after SABRE-hyperpolarised measurements have been performed. Note that no acrolein signals are visible in b). Signals denoted 'Im', 'C' and 'CO' refer to imidazole, the IMes ligand of the SABRE catalyst and cyclooctane (which is formed after catalyst activation) respectively. Unassigned resonances belong to other molecules present in the electronic cigarette aerosol solution (see Section S2).*

S4.2 SABRE Hyperpolarised NMR of Ecig^{diluent}

A solution containing [IrCl(COD)(IMes)] (5 mM) (where COD is *cis*,*cis*-1,5-cyclooctadiene and IMes is 1,3-bis(2,4,6-trimethyl-phenyl)imidazol-2-ylidene) and imidazole (15 equiv.) in methanol-d_4 (0.5 mL) was activated with H_2 (3 bar) for a few hours at room temperature. At this point 50 µL of the electronic cigarette aerosol solution was added before pH_2 shaking was performed (3 bar for 10 seconds at 6.5 mT). Later, a second 50 µL addition of the electronic cigarette aerosol solution was performed. SABRE-hyperpolarised spectra are shown in Figure S15. A time course for the hyperpolarised acrolein signals is shown in Figure S16.

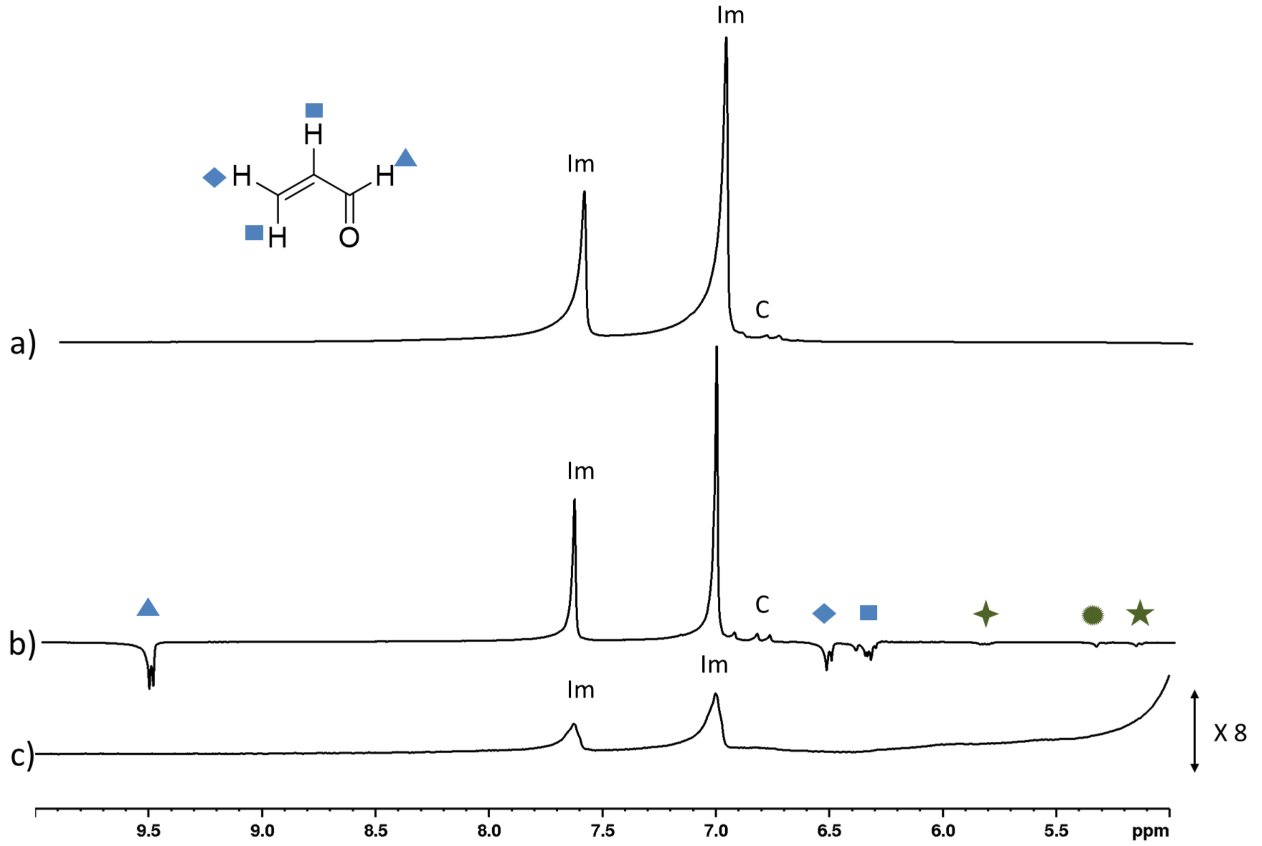

***Figure S15:** Partial single scan ^{1}H NMR spectra recorded at 9.4 T and 298 K of a) [IrCl(COD)(IMes)] (5 mM) and imidazole (15 equiv.) in 0.5 mL methanol-d_4 shaken with 3 bar pH_2 for 10 seconds at 6.5 mT. b) SABRE HP spectra of the solution used in a) ca 5 minutes after addition of 50 µL Ecig^{diluent}. c) Thermally polarised ^{1}H NMR spectrum of the solution used in b) ca 10 minutes after the addition of Ecig^{diluent} to the solution used in a). Note that signals denoted 'Im' and 'C' refer to imidazole and the IMes ligand of the SABRE catalyst respectively and that c) is expanded vertically by a factor of 8 relative to a) and b). Those signals marked by the green symbols are consistent with 1,4-pentadien-3-ol or related molecules.*

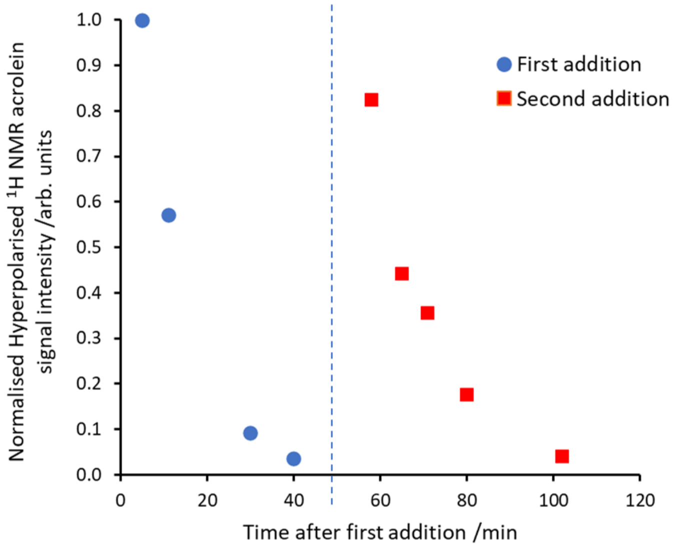

***Figure S16:** Time course of hyperpolarised acrolein signals after two 50 µL portions of Ecig^{diluent} were added to a solution of [IrCl(COD)(IMes)] (5 mM) and imidazole (15 equiv.) in 0.5 mL methanol-d_4. Each data point involves shaking the solution with fresh 3 bar pH_2 for 10 seconds at 6.5 mT. The vertical dashed line indicated the point at which a further portion of electronic cigarette aerosol solution was added.*

Estimates of hyperpolarised T_1's of acrolein could be determined by recording a series of single scan SABRE-hyperpolarised ^{1}H NMR spectra with 10 degree flip angles. The decay of hyperpolarised signal intensity over time is shown in Figure S17 and occurs due to radiofrequency excitation and nuclear spin relaxation. This can be accounted for using the following equations to extract a T_1.

In this model the hyperpolarized signal of species A, $(S_A)_t$, detected by the 10° pulse at time t is calculated according to Equation 1, where $(M_A)_t$ is the magnetization of species A at time t and θ is the flip angle. The magnetization of species A remaining after the pulse is given by Equation 2. The magnetization of A changes during the time interval between successive pulses according to nuclear spin relaxation, as described in Equation 3. Values of $(M_A)_t$ were calculated from the model and compared to values determined from experiments. T_1 was found by minimising the least squared summed differences between experimental and calculated values. Similar approaches have been used elsewhere.[12–14]

$$(S_A)_t = (M_A)_{t-\delta t} sin\theta \quad (1)$$

$$(M_A)_t = (M_A)_{t-\delta t} cos\theta \quad (2)$$

$$(M_A)_{t+\delta t} = (M_A)_t - \left(\frac{(M_A)_t}{T_1}\right)\delta t \quad (3)$$

In this example the model gives only an estimate for T_1 as it does not account for decay of hyperpolarised signals due to chemical reaction (such as binding to the metal centre, hydrogenation or other decomposition). This should be a valid assumption as the reaction rate is expected to be significantly slower than the relaxation rate. (*i.e.* reaction under these conditions occurred over a *ca* 40 minute time window, see Figure S16), therefore within this 40 second observation window the dominant processes leading to signal decay that must be accounted for are radiofrequency excitation and relaxation and chemical reaction has therefore been omitted.

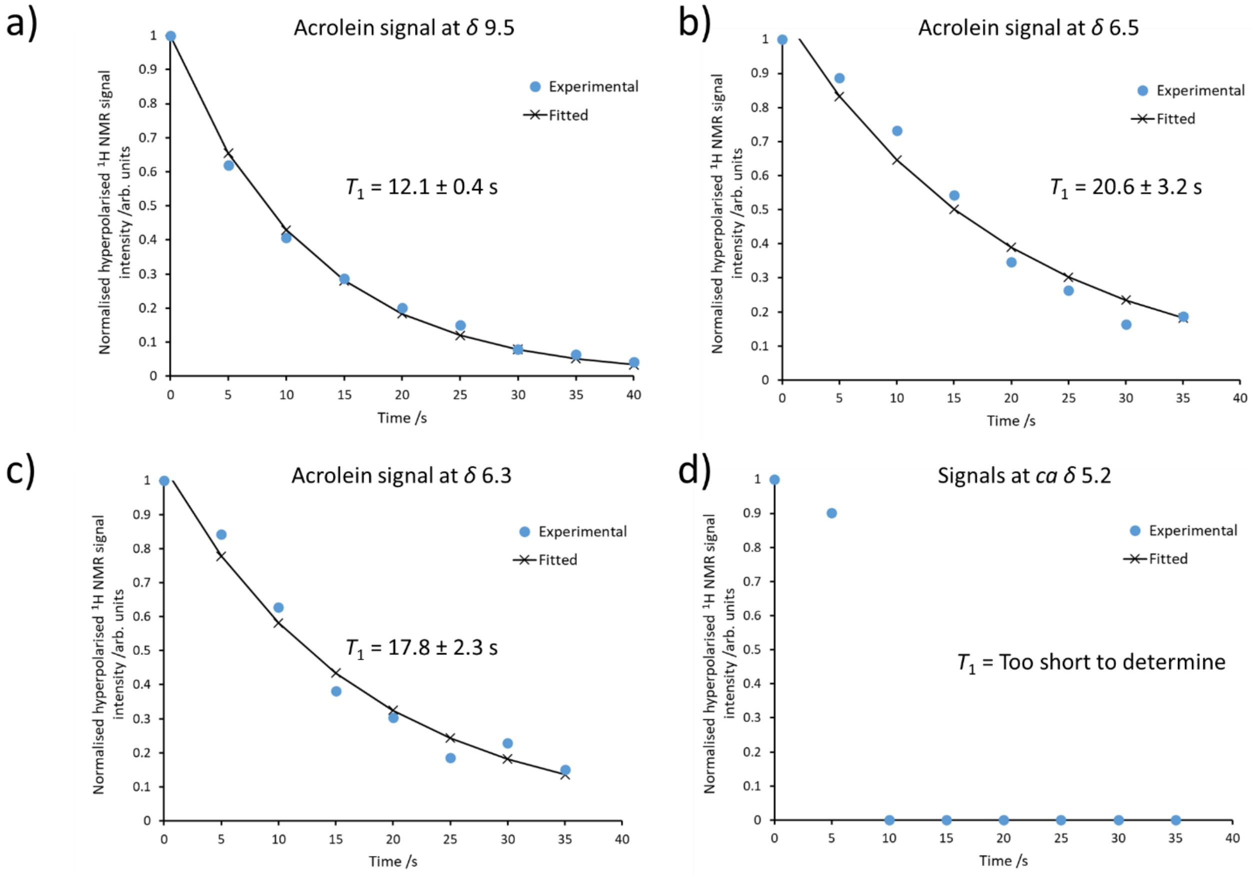

Figure S17: Decay of hyperpolarised acrolein signals as a function of time recorded using a series of single scan SABRE-hyperpolarised ^{1}H NMR spectra with 10° flip angles. These spectra were recorded following shaking a solution of [IrCl(COD)(IMes)] (2 mM), imidazole (15 equiv.) and Ecig^{diluent} solution (50 µL) in 0.5 mL methanol-d_4 with 3 bar pH_2 for 10 seconds at 6.5 mT. These spectra were collected ca 5 minutes after addition Ecig^{diluent}. Note that the experimentally determined points used to fit the T_1 are shown by the marker points and the connecting line is only to aid visual interpretation.

S5. References

1 I. Kownacki, M. Kubicki, K. Szubert and B. Marciniec, *J. Organomet. Chem.*, 2008, **693**, 321–328.

2 R. W. Adams, J. A. Aguilar, K. D. Atkinson, M. J. Cowley, P. I. P. Elliott, S. B. Duckett, G. G. R. Green, I. G. Khazal, J. López-Serrano and D. C. Williamson, *Science*, 2009, **323**, 1708–1711.

3 K. M. Appleby, R. E. Mewis, A. M. Olaru, G. G. R. Green, I. J. S. Fairlamb and S. B. Duckett, *Chem. Sci.*, 2015, **6**, 3981–3993.

4 L. S. Lloyd, A. Asghar, M. J. Burns, A. Charlton, S. Coombes, M. J. Cowley, G. J. Dear, S. B. Duckett, G. R. Genov and G. G. R. Green, *Cat. Sci. Technol.*, 2014, **4**, 3544–3554.

5 M. Fekete, P. J. Rayner, G. G. R. Green and S. B. Duckett, *Magn. Reson. Chem.*, 2017, **55**, 944–957.

6 M. Fekete, S. S. Roy and S. B. Duckett, *Phys. Chem. Chem. Phys.*, 2020, **22**, 5033–5037.

7 S. Knecht, S. Hadjiali, D. A. Barskiy, A. Pines, G. Sauer, A. S. Kiryutin, K. L. Ivanov, A. V Yurkovskaya and G. Buntkowsky, *J. Phys. Chem. C.*, 2019, **123**, 16288–16293.

8 W. H. Duckworth, *Improving NMR Sensitivity: The Synthesis and SABRE Evaluation of Nicotine Isotopologues*, 2018, University of York, PhD Thesis.

9 N. Eshuis, R. L. E. G. Aspers, B. J. A. van Weerdenburg, M. C. Feiters, F. P. J. T. Rutjes, S. S. Wijmenga and M. Tessari, *Angew. Chem. Int. Ed.*, 2015, **54**, 14527–14530.

10 N. Eshuis, B. J. A. van Weerdenburg, M. C. Feiters, F. P. J. T. Rutjes, S. S. Wijmenga and M. Tessari, *Angew. Chem. Int. Ed.*, 2015, **54**, 1481–1484.

11 N. Eshuis, N. Hermkens, B. J. A. van Weerdenburg, M. C. Feiters, F. P. J. T. Rutjes, S. S. Wijmenga and M. Tessari, *J. Am. Chem. Soc.*, 2014, **136**, 2695–2698.

12 B. J. Tickner, P. J. Rayner and S. B. Duckett, *Anal. Chem.*, 2020, **92**, 9095–9103.

13 P. J. Rayner, P. M. Richardson and S. B. Duckett, *Angew. Chem.*, 2020, **132**, 2732–2736.

14 M. E. Merritt, C. Harrison, Z. Kovacs, P. Kshirsagar, C. R. Malloy and A. D. Sherry, *J. Am. Chem Soc.*, 2007, **129**, 12942–12943.
